# Turnover intention among intensive care nurses and the influence of the COVID-19 pandemic: a scoping review

**DOI:** 10.1186/s12960-025-00992-7

**Published:** 2025-05-15

**Authors:** Tanja Lesnik, Anna Hauser-Oppelmayer

**Affiliations:** 1 University of Klagenfurt, Department of Public, Nonprofit & Health Management, Universitätsstraße 65-67, 9020 Klagenfurt, Austria; 2 Carinthia University of Applied Sciences, Villach, Austria

**Keywords:** Intention to leave, Intention to stay, Intensive care, Nurses, Scoping review

## Abstract

**Background:**

The shortage of nurses has been an ongoing issue for many decades. An important contributing factor is voluntary turnover. Especially in intensive care (ICU) and critical care units (CCU) with high workloads, high mortality rates and stressful working conditions, the phenomenon has serious consequences. In addition, the COVID-19 pandemic has exacerbated the problem. This review examines the factors influencing the intention to leave (ITL) and intention to stay (ITS) among intensive care and critical care nurses and the influence of the COVID-19 pandemic.

**Methods:**

A scoping review was conducted based on the Preferred Reporting Items for Systematic Reviews and Meta-Analyses extension for Scoping Reviews (PRISMA–ScR). The databases PubMed, Wiley, Scopus, APA PsycNet and Web of Science were searched. In addition, a forward search using Google Scholar was carried out. Empirical studies reporting on factors influencing the intention to stay or leave among ICU nurses published from 2000 to 2022 were included. The factors were qualitatively coded in MAXQDA, resulting in an inductive coding frame.

**Results:**

Fifty-four studies, including 51 quantitative, one qualitative, and two mixed methods studies, were included in the review. The analysis of factors influencing the intention to either leave or stay in intensive care can be systematically classified into two categories: organisational factors and individual factors. The category of organisational factors encompasses factors, such as commitment and integration, leadership, professional collaboration and communication. Conversely, the category of individual factors comprises factors, such as professionalism, job satisfaction, mental health and social reasons. The pandemic has exacerbated certain aspects within individual and organisational factors, influencing the intention to leave intensive care. Notably, despite the significant impact of COVID-19, no “new” themes are directly attributable to it.

**Conclusions:**

The results can help practitioners meet future challenges (maintaining adequate staffing levels in view of the existing shortage of nurses). It is the responsibility of nursing and hospital management to capitalise on the insights of this review. Future research should focus on longitudinal, interventional and qualitative study designs to understand voluntary turnover among ICU nurses.

**Supplementary Information:**

The online version contains supplementary material available at 10.1186/s12960-025-00992-7.

## Background

The shortage of nurses has been an ongoing issue for many decades and poses significant challenges to healthcare systems worldwide [1]. The lack of nurses in intensive and critical care units (ICU) is particularly problematic due to an elevated nurse-to-patient ratio and the requirement for specialised nursing skills [2]. Furthermore, physically, ethically, and psychologically these units are highly demanding working areas [3]. The COVID-19 pandemic has placed additional pressure on ICU staff due to repeated waves of the disease and the large number of critically ill patients [4].

A significant factor contributing to the healthcare workforce shortage in intensive care units is voluntary turnover [5], in contrast to involuntary turnover, for example, through retirement or illness [6].

Building on turnover theory [5, 7], this paper focuses on voluntary turnover intention, the status preceding actual turnover. This intention can be explained as the conscious desire to leave an organisation voluntarily before actually leaving. Therefore, this plays an essential role in turnover research [6]. Nurses’ intention to leave the ICU has received increasing attention in the scientific literature in recent years. As a result, a majority of studies have examined this phenomenon by directly or indirectly linking several variables to the intention to leave (ITL) or the intention to stay (ITS) [8–12]. However, to understand the problem in its entirety, a comprehensive view of the different influencing factors is needed.

This review aims to identify and examine the factors contributing to turnover intention among intensive care nurses while concurrently assessing the impact of the COVID-19 pandemic on the intention to leave the intensive care workplace.

## Methods

### Review design

This scoping review was reported based on the Preferred Reporting Items for Systematic Reviews and Meta-Analyses extension for Scoping Reviews (PRISMA–ScR) checklist (see Additional file 3) [13]. The executed review was guided by an a priori study protocol, as shown in Additional file 1, and examined secondary data from five different data bases.

Originally, we planned to conduct a systematic literature review. However, Munn et al. [14] argue that if a review is more interested in the identification and synthesis of major characteristics (influencing factors) in studies and in the mapping, reporting or discussion of these characteristics, then a scoping review approach is the better option. This finding is in line with the methodological framework of scoping reviews of Arksey and O’Malley [15] and the instructions for conducting a scoping review of the JBI Manual for Evidence Synthesis [16], which we used to guide our review. In addressing our research questions, it was imperative to encompass all pertinent studies, irrespective of their methodological approaches.

### Search strategy

In the formulation of the research questions, alongside the delineation of the study’s objectives and the establishment of eligibility criteria, the study employed the PCC (population, concept, context) framework, as outlined in Table 1.

**Table 1:** Eligibility criteria

	Inclusion criteria	Exclusion criteria
Population	Nurses	Other health care professionals
Concept	Intention to leave (ITL), intention to stay (ITS) according to Hom et al. [20]	Actual leaving, actual staying
Context	Neonatal, pediatric, and adult Intensive Care Unit (ICU), Critical Care Unit (CCU) in hospital setting	Intensive Care Unit (ICU) or Critical Care Unit (CCU) in the outpatient setting
**Keywords used in database search** Intent* to leave OR intent* to quit OR turnover intent* OR intent* to stay AND critical care OR intensive care AND nurs*		

A comprehensive database search of the Wiley, Web of Science, Scopus, PubMed and APA Psycnet databases was conducted separately by both authors from September 2022 to February 2023. The keywords used were defined in advance and tested in the databases. The defined keywords (Table 1) were combined using advanced fieldcode searching (TITLE–ABS–KEY), phrase searching, truncation, and Boolean operators "OR" and "AND" (Additional file 2). To identify further records a forward search was carried out via Google Scholar. A date restriction from 2000 to 2022 was applied, because a significant shortage of nurses was projected from the turn of the millennium onward [17].

### Study selection

Microsoft Excel was used to make the documentation of our literature search transparent and comprehensive in every step. All authors participated in the identification and selection of the studies. The research team assessed the potential relevance of the included studies after reading the abstract and the full text. In the event of unresolved conflicts in the screening process, there was the option to consult a third reviewer. There was no need for consultation, as all differences were reconciled within the research team after the first round of discussion.

### Eligibility criteria

Considering the existence of two prior publications addressing this subject matter [18, 19], our research methodology was predicated on a thorough examination of the keywords and eligibility criteria identified within these articles. This approach was complemented by a structured brainstorming session, an extensive review of the existing literature, and a preliminary piloting phase. The piloting of the keywords was undertaken separately by both authors, with the finalised selection of keywords being the result of a collaborative discussion and refinement process within the research team. To select studies relevant to our study objective we applied the eligibility criteria depicted in Table 1. In addition, we applied the following inclusion criteria:

- Peer reviewed articles
- Articles written in English language
- Qualitative, quantitative, mixed methods, experimental or quasi experimental methodological designs
- All articles must have been published between 2000 and 2022

### Data extraction

We extracted data from the final set of 54 incorporated articles, including the author’s name, country, sample size, type of ICU setting, study design, instruments, objective, and variables used. Microsoft Excel was used to extract the data. The variables that displayed a correlation with ITL or ITS or that were described narratively in qualitative papers were included for data analysis. We analysed the data using MAXQDA software, which resulted in an inductive [15] coding framework (see Additional file 4).

### Data synthesis

The variables exported into MAXQDA were categorised into organisational and individual factors associated with ITL or ITS. We also developed another category in our coding framework to capture the impact of COVID-19 on the ITL. Similar explanatory factors were grouped into subcategories. The categories emerging from the extraction process are shown in Figs. 2, 3, 4, 5, 6 in the "Results" section. Both researchers extracted the data and were involved in the inductive categorisation process to ensure objectivity. In the event of unresolved conflicts, a third reviewer was consulted. However, as there were only a few differences in the codes, indicating almost perfect agreement, there was no need to consult the third reviewer (Table 2).

**Table 2:** Study characteristics overview

**Methodology**	
Quantitative	51
Qualitative	1
Mixed-methods	2
**Geographical distribution**	
Europe	17
America	15
Europe and US	1
Middle East	14
Asia–Pacific	5
No geographic context mentioned	2
**Sample size (quantitative and mixed-methods studies)**	
Smallest sample size in the included studies	40
Largest sample size in the included studies	5824
**Sample size (qualitative study)**	10
**Publication year**	
Before 2020	28
During COVID-19 pandemic 2020–2022	26
**Studies measuring a COVID-19 impact on turnover intentions**	8

## Results

### Search results

**Fig. 1:**
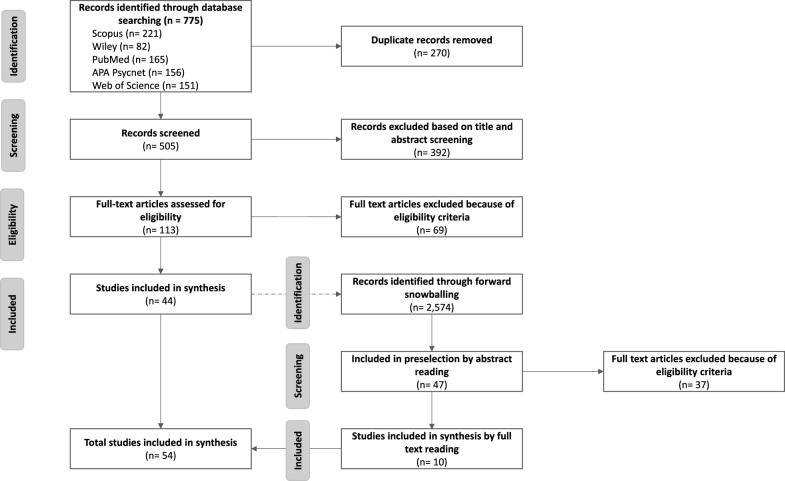
PRISMA–ScR flowchart of the database search and study selection

From 775 studies identified through database search, we excluded 270 duplicates and reviewed the titles and abstracts of 505 remaining records. Of these, we identified 113 articles, on which we conducted a full-text screening. Of these 113 studies, 44 met our eligibility criteria and were included. A systematic forward search conducted on these 44 records through Google Scholar yielded 2574 potential articles. Subsequent screening based on abstracts and full texts resulted in the selection of 47 articles. Ultimately, a rigorous selection process facilitated the inclusion of 10 additional articles for the final analysis. The PRISMA–ScR flow diagram in Fig. 1 reports the screening process and depicts the numbers of records identified, articles excluded, and studies included.

In total, we included 54 studies in our final synthesis (see Table 3) [3, 8–12, 21–68].

**Table 3:**
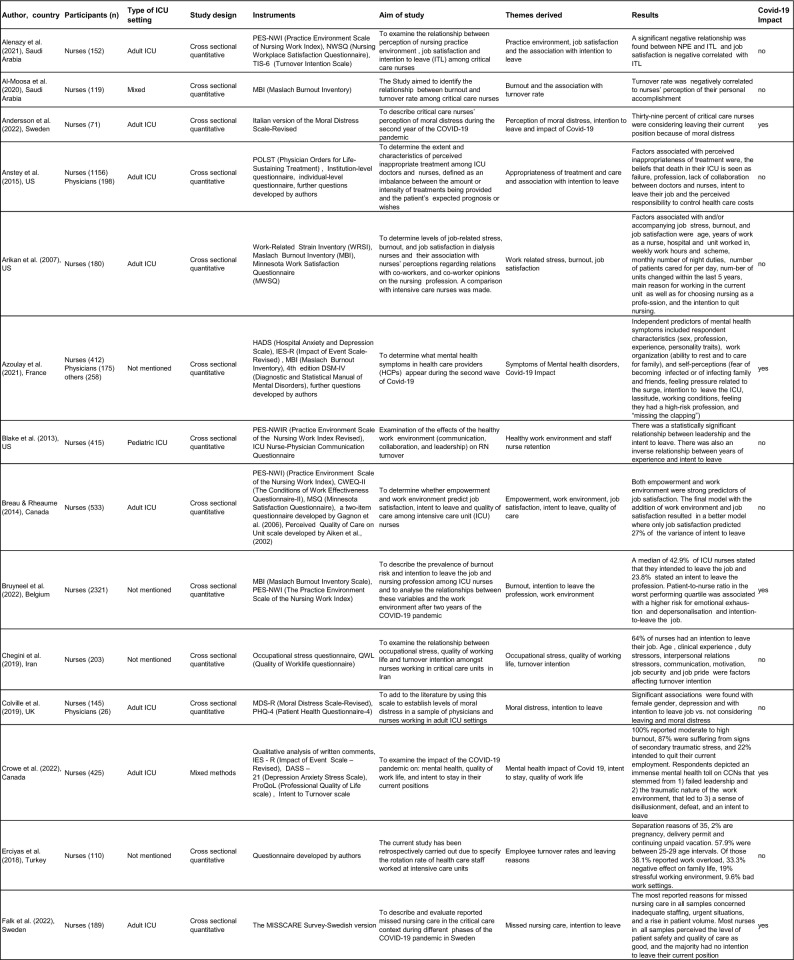

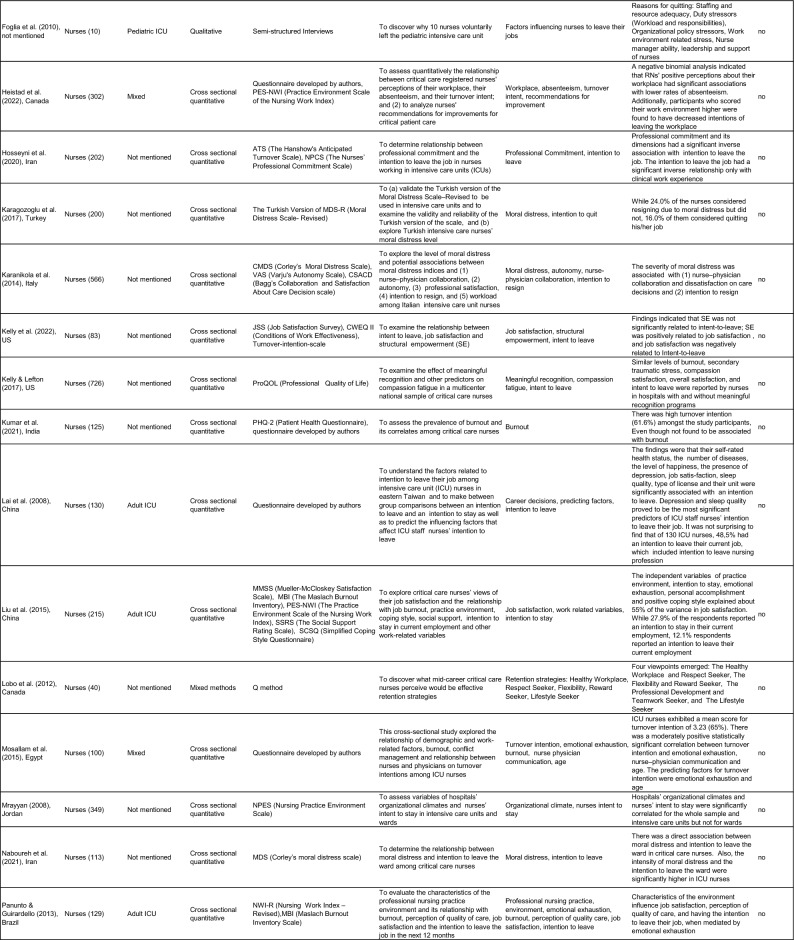

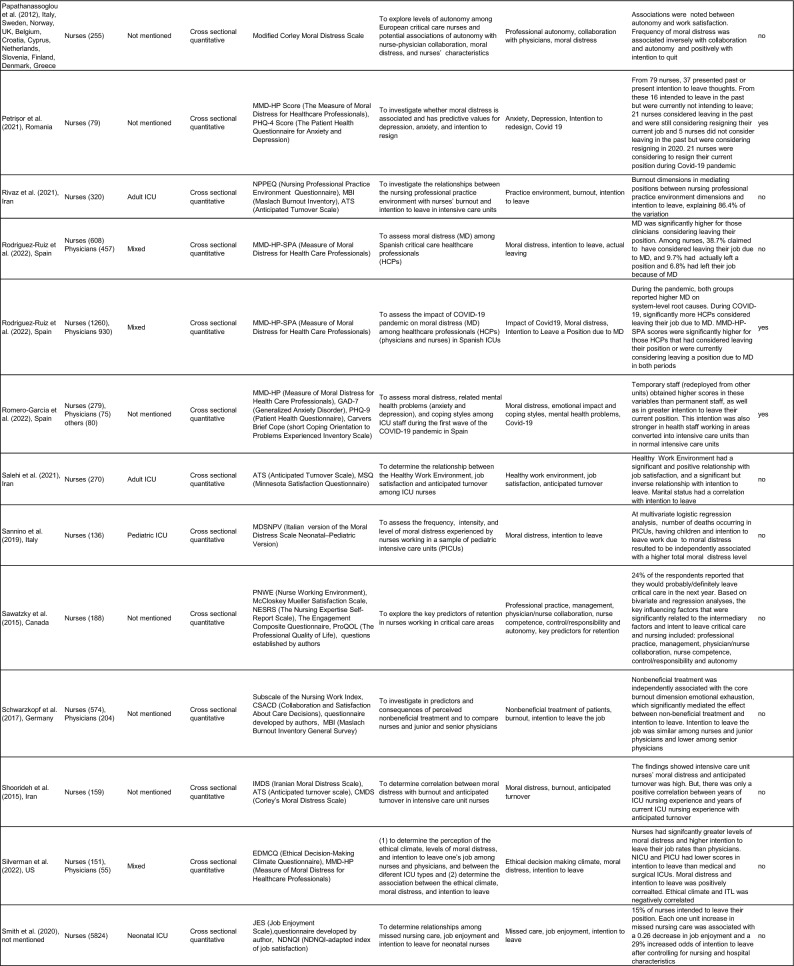

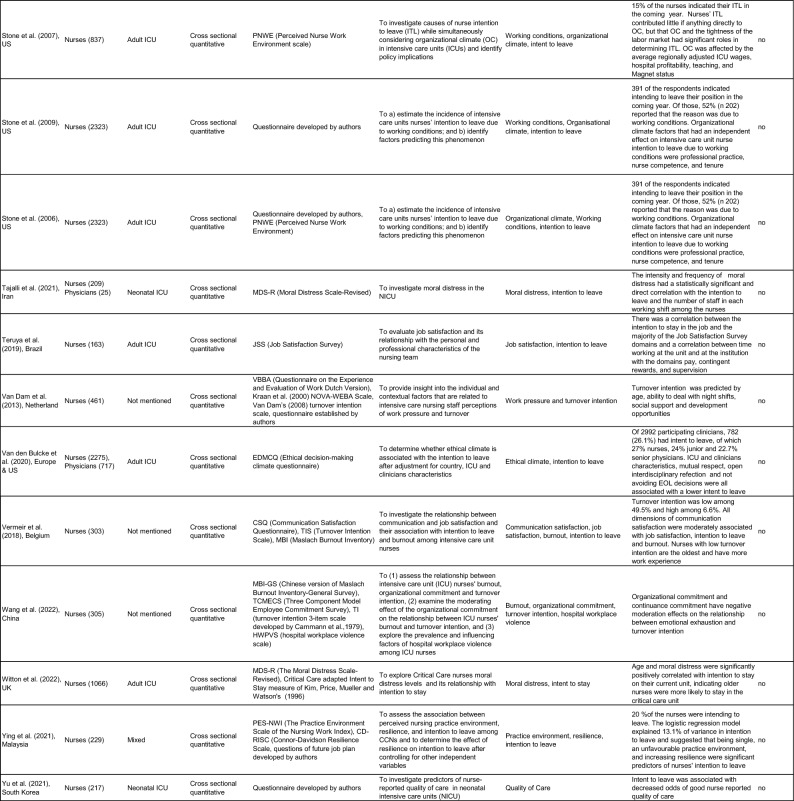
Study characteristics, results and themes derived

### Analysis results

Our analysis yielded two main categories, organisational and individual factors regarding the intention to leave and the intention to stay in intensive care. Organisational factors contributing to ITL encompass factors, such as (1) commitment and integration, (2) quality of delivered care, (3) organisational structure and work environment, (4) leadership, and (5) professional collaboration and communication, whereas organisational factors contributing to ITS exclude (1) commitment and integration. Individual factors contributing to ITL encompass factors, such as (1) mental health and social reasons, (2) socio-demographic characteristics, (3) professionalism, (4) job satisfaction and (5) internal drivers, whereas individual factors contributing to ITS are limited to (1) professionalism and (2) internal drivers. Organisational COVID-19-related factors associated with ITL encompass factors, such as (1) quality of delivered care and (2) organisational structure and work environment. Individual COVID-19-related factors associated with ITL include mental health and social reasons.

While the majority of studies focused on ITL, only five studies investigated the ITS [38, 41, 43, 54, 62]. This distinction should be noted, as the literature emphasises that the factors leading to ITL cannot be inversely interpreted as factors leading to ITS [69].

### Organisational factors

**Fig. 2:**
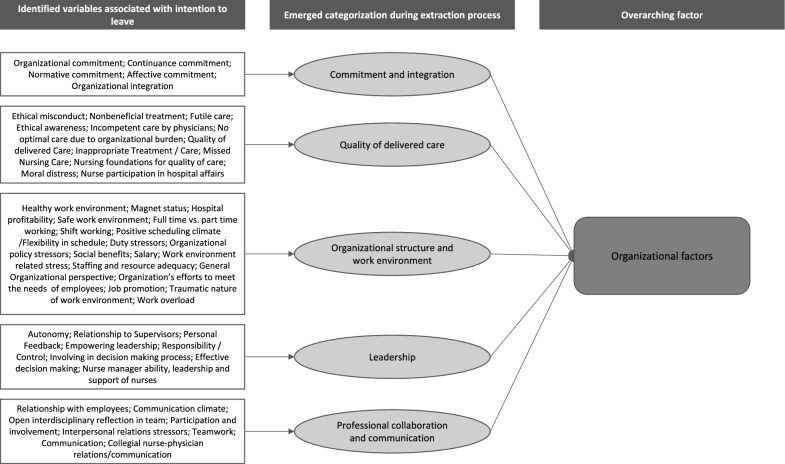
Organisational factors associated with ITL

As shown in Fig. 2, the identified variables associated with organisational influences were grouped into five categories. Commitment and integration refer to personal commitment to the organisation, where, for example, Wang et al. [65] found essential correlations with intention to leave among ICU and CCU nurses.

Furthermore, several studies have shown that moral distress [29, 36, 53], nonbeneficial treatment [55] and quality of delivered care [28, 45, 68] are associated with increased turnover intention. The organisational structure and work environment include the leadership and support of nurses [12, 28, 48], the work environment [40], and the organisational structure [8]. In the category of professional collaboration and communication, variables such as collegial nurse–physician communication [10, 54, 60], teamwork [57], and communication climate were subsumed [64].

**Fig. 3:**
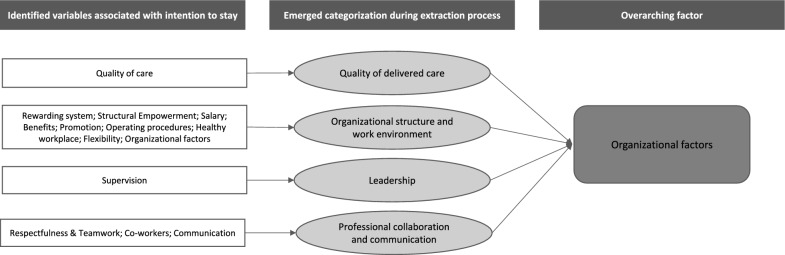
Organisational factors associated with ITS

Similar to the findings for ITL, organisational factors were also significantly related to ITS. Four categories emerged during the extraction process. These include quality of delivered care [43] or organisational structure and work environment. ICU and CCU nurses who perceived high rewards [41, 62] were, for example, more prone to consider staying. Additional variables were assigned to leadership, professional collaboration, and communication (e.g., the importance of teamwork) [41] (Fig. 3).

### Individual factors

Individual factors contributing to ITL encompass (1) mental health and social reasons, (2) socio-demographic characteristics, (3) professionalism, (4) job satisfaction, and (5) internal drivers.

**Fig. 4:**
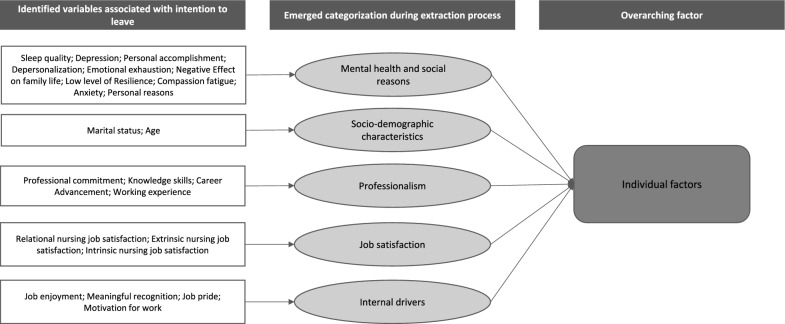
Individual factors associated with ITL

Various variables related to nurses’ individual characteristics associated with their ITL were identified. Figure 4 shows that most of the variables were related to mental health and social factors, such as depression [39, 40] and resilience [67]. Other categories that correlate with ITL include socio-demographic characteristics [52], professionalism [43], job satisfaction [37, 45], and internal drivers, for example, motivation for work [8].

In terms of individual factors related to the intention to stay, professional development [41] and high levels of compassion satisfaction [38] were found to be related to ITS. In a study conducted by Kelly et al. [38] high levels of compassion satisfaction related to ITS were found.

**Fig. 5:**
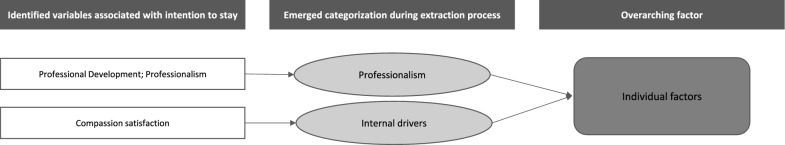
Individual factors associated with ITS

Overall, the mapping of the identified variables showed that organisational factors are predominant in ICU nurses’ intention to leave or stay (Fig. 5).

### ITL during the COVID-19 period

**Fig. 6:**
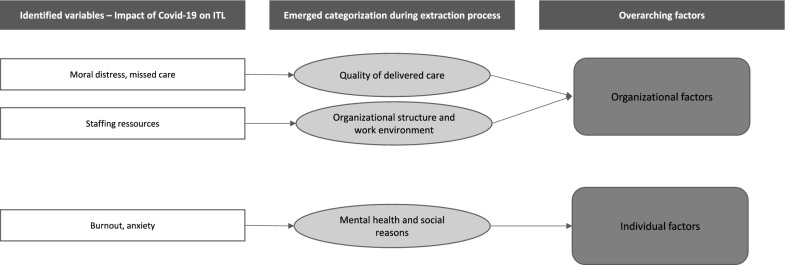
COVID-19-related factors associated with ITL

The pandemic enhanced problems related to organisational structure and the work environment and placed additional pressure on ICU and CCU nurses. In addition, the existing staff shortage in ICU was further exacerbated by the pandemic [32]. The analysis showed that mental health problems (e.g., burnout, anxiety) occurred more frequently during COVID-19 than they did in the prepandemic period [3, 26, 47] and led to ITL. Studies from Romania [47] and Spain [49] have shown that more nurses reported a perception of moral distress during the pandemic and their ITL increased. Falk et al. [32] reported an adverse correlation between missed nursing care and ITL due to COVID-19 (Fig. 6).

## Discussion

This review identified 54 studies of nurses’ intention to leave or stay in intensive and critical care settings from 23 countries in four regions and synthesised the study results. The findings of the study identified multiple factors influencing ITL or ITS among nurses in the ICU/CCU and highlighted the impact of COVID-19 on ITL.

Recent research by Khan et al. [19] revealed similar organisational factors affecting the ITL of ICU nurses, but did not consider factors influencing the ITS. The present review focuses on the factors that enhance the ITS among intensive care nurses. Moreover, it shows that particular emphasis was placed on inadequate staffing resources in intensive and critical care, which contributes to intensive care nurses’ ITL [3, 10, 12, 48, 60]. In addition, our results (see Fig. 6) are in line with the findings of Xu et al. [18], who have reported on the importance of nursing leadership and its influence on ITL. Thus, the leadership and management support of the nursing staff should be addressed in nursing management to reduce turnover intentions among ICU nurses. In contrast to normal ward nurses, where Marques-Pinto et al. [70] found no correlation between nurse–physician collaboration and ITL, teamwork and interprofessional collaboration in the intensive care setting play a major role in considering leaving the position [47, 55, 57, 64]. This was also discussed in the preliminary work of Khan et al. [19]. Strengthening nurses’ autonomy [54] and relationships within teams are essential factors in tackling the turnover intention of intensive care nurses [57]. Interestingly, this review identified only four studies that reported inadequate financial remuneration as a contributing factor to ITL (see, for example [60]) or adequate remuneration contributing to ITS (see, for example [62]). Although remuneration is a contributing factor, organisational structures such as autonomy [57], leadership [12, 33], or executing optimal care without pressure due to inadequate staffing resources [53, 57, 58] had more impact on ITL and ITS among ICU nurses.

Individual factors that often lead to mental health problems due to the work environment, such as moral distress at the workplace [36] or ethical misconduct [23], were the predominant findings at the individual level.

Studies analysing the impact of the COVID-19 pandemic on turnover intention have shown that the pandemic has had an amplifying effect on the already existing problems, particularly regarding the organisational structure and work environment, e.g., moral distress [49, 51], and missed nursing care due to long working hours and inadequate staffing resources [32]. Interestingly, no "new" themes directly related to COVID-19 could be identified. However, unresolved problems have deteriorated further, and particularly during the pandemic intensive and critical care units have not been sufficiently staffed for this additional challenge [32].

## Limitations

Due to cultural, health system, and workplace differences between the regions and the heterogeneity in study objectives, designs, and instruments used, generalisations cannot be made. We acknowledge that by not conducting a quality assessment while applying the scoping review methodology, we have introduced additional limitations. Thus, an effort was made to address the quality of the studies by applying the eligibility criteria "peer-reviewed articles only". In addition, a possible reporting bias cannot be excluded based on secondary data analysis.

## Conclusion

This review provides a global and multifaceted overview of the factors contributing to the ITL and ITS of intensive care nurses, including the impact of COVID-19 on ITL. The results highlight the influence of organisational factors (inadequate staffing levels, inappropriate leadership) on turnover intention. Our findings can help practitioners meet future challenges (the increasing demand for healthcare services due to the aging population, or maintaining adequate staffing levels in view of the already existing shortage of nurses). It is the responsibility of nursing- and hospital management to capitalise on these insights. Future research should focus on longitudinal, qualitative, and interventional studies in different cultural contexts and health systems to gain a better understanding of the phenomenon of voluntary turnover.

## Supplementary Information

Additional file 1: Study protocol.

Additional file 2: Search strategy and search terms.

Additional file 3: PRISMA-ScR checklist.

Additional file 4: Coding framework.

## Data Availability

Data are available from the corresponding author upon request.

## Abbreviations

ITL: Intention to leave
ITS: Intention to stay
ICU: Intensive Care Unit
CCU: Critical Care Unit
COVID-19: Coronavirus disease 2019
PRISMA–ScR: Preferred reporting items for systematic reviews and meta-analyses extension for scoping reviews
PCC: Population, concept, context
