# Supplementary material for: Turnover intention among intensive care nurses and the influence of the COVID-19 pandemic: a scoping review
Source: Hum Resour Health. 2025 May 15;23:23. doi: 10.1186/s12960-025-00992-7 (PMC12080060; doi:10.1186/s12960-025-00992-7)
Supplement: Supplementary file 1 — Additional file 1: Study protocol. [file 12960_2025_992_MOESM1_ESM.docx]

**Additional file 1** Study protocol

**Factors influencing turnover intention among intensive care nurses and the influence of the Covid-19 pandemic: A systematic literature review**

Tanja Lesnik^1^ & Anna Hauser-Oppelmayer^2^

^1^University of Klagenfurt, Department of Public, Nonprofit & Health Management, Universitätsstraße 65-67, 9020 Klagenfurt, Austria. E-Mail: [tanja.lesnik@aau.at](mailto:tanja.lesnik@aau.at), phone number: +43 463 2700 4138. ORCID: 0009-0001-9981-3129. Corresponding author.

^2^University of Klagenfurt, Department of Public, Nonprofit & Health Management, Universitätsstraße 65-67, 9020 Klagenfurt, Austria. E- Mail: [anna.oppelmayer@aau.at](mailto:anna.oppelmayer@aau.at), phone number: +43 463 2700 4136. ORCID: 0000-0002-3572-3589.

**Introduction and relevance**

The shortage of nurses has been an ongoing issue for many decades and poses significant challenges to healthcare systems worldwide (1). In particular, the lack of nurses in intensive and critical care units (ICU/ CCU) is problematic due to an elevated nurse-to-patient ratio and the requirement for specialized nurses (2). Furthermore, these units are physically, ethically, and psychologically highly demanding working areas (3). The Covid-19 pandemic placed additional stress on ICU staff due to repeated waves of the disease and a large number of critically ill patients (4).

A significant contributing factor to the healthcare workforce shortage is voluntary turnover (5), in contrast to involuntary turnover, for example, through retirement or illness (6). Building on turnover theory (7, 5), this paper focuses on voluntary turnover intention, the status before actual turnover. This intention can be explained as the conscious wilfulness to leave an organization voluntarily before actually leaving and is therefore playing an essential role in turnover research (6). Nurses’ intention to leave the ICU has received increasing attention in the scientific literature in recent years. Thereby, a majority of studies examined the phenomenon by linking several variables directly or indirectly to intention to leave (ITL) or intention to stay (ITS) (8–12). However, to understand the problem in its entirety, a comprehensive view of the different variables is needed.

**Objective and Research Questions**

**Objective:** The systematic literature review aims to explore the factors influencing critical care nurses' turnover intention and the impact of the Covid-19 pandemic on their intention to leave or stay.

**RQ 1:** What factors impacting intensive and critical care nurses' intention to leave have been identified by the scientific literature so far?

**RQ 2:** What factors impacting intensive and critical care nurses' intention to stay have been identified in the scientific literature so far?

**RQ3:** What findings did the literature reveal regarding the impact of the COVID-19 pandemic on critical care nurses' intention to leave or stay?

**Protocol**

The study protocol for this systematic literature review was prepared in autumn 2022 to capture the systematic approach of the planned systematic literature review on "Factors influencing turnover intentions of critical care nurses and the impact of the Covid-19 pandemic". The review was guided by the PRISMA guideline and checklist. The study protocol was not registered. The study protocol acts as a guide for both researchers to follow the same structure and systematic approach and to increase transparency of the planned approach.

**Study design**

This systematic review will focus on factors influencing turnover intention (intention to leave and intention to stay) of intensive and critical care nurses and the impact of the Covid-19 pandemic on intention to leave or stay. The review will include qualitative and quantitative, mixed-methods, experimental, quasi-experimental, cross-sectional and longitudinal studies. Systematic reviews, meta-analyses and other non-empirical articles will be excluded.

**Population**

We only include studies that examine the phenomenon of turnover intention among intensive and critical care nurses. If the occupations in the studies are mixed, we only include them if > 50% of the study population are intensive and critical care nurses. This must be explicitly stated in the studies. Moreover, we only include studies that show that more than 50% of the settings studied are intensive and critical care settings.

**Topic description with adapted PICO(S) criteria**

The PICO(S) scheme is used to identify the objective of the applied research questions. The PICO(S) elements form the basis for the eligibility criteria, which were established after identifying the key elements of the review using the PICO(S) scheme. Originally, the PICO(S) scheme was developed for evidence based medicine (13). But also used in psychology and recommended by the Cochrane Library (14). The key elements of the PICO(S) scheme are presented in the following section.

| **PICO(S) Criteria** |  |
| --- | --- |
| **P**opulation | Intensive and critical care nurses |
| **I**ndicator/ Intervention | Not applicable |
| **C**ontext (Comparator, Control) | Critical care/ intensive care setting (acute hospitals) |
| **O**utcome | factors influencing intention to leave/ intention to stay / turnover intention |
| **S**tudy design | observational studies (qual + quant), quasi-experimental studies, experimental studies, cross sectional studies, longitudinal studies, mixed-methods studies |

**Eligibility criteria**

| **Databases** |
| --- |
| PUBMED, WILEY, SCOPUS, APA PSYCNET, WEB OF SCIENCE |
| **Key search terms** |
| intent* to leave OR intent* to quit OR turnover intent* OR intent* to stay AND critical care OR intensive care AND nurs* |
| **Inclusion criteria** |
| Intention to quit, intention to leave, intention to stay (turnover antecedents)  Peer-reviewed articles between 2000-2022  Language: English  Intensive care/ critical care setting of adults and children (adult, paediatric and neonatal)  > 50% of study participants are intensive/ critical care frontline nurses  Qualitative, quantitative or mixed methods exploratory approaches and experimental or quasi-experimental designs  > 50% intensive care units or critical care units |
| **Exclusion criteria** |
| non-peer-reviewed articles  other languages than English  published before the year 2000  non-hospital settings  other units (e.g. normal wards, perioperative wards)  < 50% of study participants are intensive/ critical care frontline nurses  Systematic reviews, Meta-Analysis, and other reviews  < 50 % intensive care units or critical care units,  < 50 % of the participants are interns, trainees or students |

**Detailed Inclusion / Exclusion criteria**

|  | **Inclusion criteria** | **Exclusion criteria** |
| --- | --- | --- |
| **Language** | English | Others |
| **Time span** | 2000-2022 | Before 2000 |
| **Locality** | Worldwide |  |
| **Keywords** | intent* to leave OR intent* to quit OR turnover intent* OR intent* to stay AND critical care OR intensive care AND nurs* | Normal wards, no intensive care nurses, nurse leaders, nursing aids, midwifes |
| **Study design** | Qualitative and quantitative designs, mixed-methods design, experimental, quasi- experimental, cross sectional studies, longitudinal studies | Systematic literature reviews, Meta-Analysis, Content analysis |
| **Abstract** | Available | Not available |
| **Ranking of journals** | Peer reviewed articles | Others |
| **Setting** | Acute Care, Hospital Setting | Others |
| **Profession** | Intensive and critical care nurses | Other professions, not mentioned profession, nursing leaders, nursing aids, midwifes |
| **Participants in studies and setting** | > 50% intensive and critical care nurses  > 50 % intensive and critical care setting | < 50% of sample  are intensive and critical care nurses, < 50% of sample are intensive and care settings |

**Outcome**

As a result of this research, the factors influencing the turnover intention of critical care and intensive care nurses and the factors influencing the intention to stay will be identified. In addition, the impact of the Covid-19 pandemic on intention to leave or stay will be identified. To be included in our review, the studies should report on either factors influencing intention to leave or factors influencing intention to stay. We also include studies that report on both outcomes. We will only include studies that report on more than 50% of critical and intensive care nurses. In addition, we will only include studies that conducted more than 50% of the examinations in ICUs. We will exclude studies that did not meet the described criteria of >50% intensive care and critical care environment and study of >50% intensive care nurses. Although it would have been interesting to also identify turnover intention of other healthcare professionals in the intensive care setting, we will only focus on nurses.

**Search strategy**

After a preliminary search in academic databases and after discussing and adapting the search strategy, we proceeded with the actual search strategy in the five databases Scopus, Pubmed, APA Psycnet, Wiley and Web of Science.

To develop the right keywords, we focused on the existing literature and also on previous keywords from papers on this topic by scholars such as Xu and colleagues (15). The research team put a lot of effort into developing the right search strategy and keywords. Due to the debate on the shortage of health professionals from the turn of the millennium onwards, we will limit the publication date to the years 2000-2022 (16, 17). We will also conduct a forward search of the included records via Google Scholar. To the best of the authors' knowledge, there are two previous reviews on this topic. A systematic literature review (18) and a meta-analysis (15), neither of which addressed the impact of Covid-19 on critical care nurses' turnover intention.

**Data management**

We will start with the literature search in the five databases mentioned. In the first search round, we will perform an abstract screening of all possible matching articles that contain our keywords in title, abstract and keywords. Before moving on to the second search round, we will exclude duplicates. In the second search round, we will screen the abstracts from the first search round and also read them in full text. In this search round, we will exclude records that do not meet our eligibility criteria. The systematic search will be conducted independently by both authors (TL and AH) to gain objectivity. After the final decision on the records screened, the research team will summarise their findings and discuss the additional articles identified by each researcher with the eligibility criteria in mind.

If no solution can be reached through discussion within the research team, a third reviewer will be consulted. In addition to the database search, a forward search will be conducted via Google Scholar. In the first round of search, we will screen the abstracts of all possible matching articles that contain our keywords in the title, abstract or keywords. In the next search round, we will sift through the included abstracts from the first search round and proceed with the full text reading. We will exclude records that do not meet our eligibility criteria. At the end of the forward search, the research team will discuss their findings. After the discussion, the number of articles agreed upon by the two researchers will additionally be included in the systematic literature review. All documentation of this search process is presented in the PRISMA diagram (19, 20).

**Assessment of study quality**

The included studies will be assessed using quality assessment tools. For quantitative observational studies the STROBE (Strengthening the reporting of observational studies in epidemiology) (21) quality assessment tool, for qualitative observational studies the COREQ (Consolidated criteria for reporting qualitative research) (22) quality assessment tool and for mixed methods studies the MMAT (Mixed methods appraisal tool) (23) quality assessment tool will be employed. The main assessment points of these tools will be methodological quality, study design, and the quality of the results and discussion section. All three quality assessment tools provide checklists for the included study designs. Risk of bias will be assessed jointly by both researchers and will be reported in the limitations section of the systematic literature review. As with the search, we will consult a third expert if there are unresolved conflicts.

**Data extraction and analysis**

We will extract data from the final set of papers, including the authors name, country, sample size, type of ICU setting, study design, instruments, objective, variables used and impact of Covid-19. Data extraction will be made with Microsoft Excel.

As a next step, we will analyze the studies using MAXQDA to develop the categories, and further synthesize the data within the research team. We will discuss all collected data from the studies in the research team and think about a categorical classification. Any conflicts or unresolved issues will be discussed in the research team. If no resolution can be reached, a third reviewer will be consulted. Assignment to the self-established categories will be done by both researchers.

**Data synthesis**

We assume that the included studies will provide heterogeneous results and will also be heterogeneous in their measurement. Therefore, we conduct data analysis and synthesis using MAXQDA software and through discussion and assignment to categories within the research team. Especially in quantitative cross-sectional studies, we expect a variety of instruments to measure the intention to leave or stay. In qualitative studies, we also expect a heterogeneous approach to data collection. Therefore, we want to summarize and assign overarching categories to the factors that influence critical care nurses' intention to leave and stay.

**Discussion**

Much valuable preliminary work on the topic of voluntary turnover and intention to leave has been done by scholars such as Griffeth, Price and Hom (24, 5). Price and Mueller (25) in particular have studied the intention to leave of normal ward nurses by using a causal model and have already described some organizational determinants. However, the literature also shows that the reasons that influence intention to stay are still not fully understood (26). Therefore, it is important to emphasise that the reasons influencing ICU nurses' intention to leave are not synonymous with the reasons influencing the intention to stay (27).
Although the phenomenon of nurses' turnover intention has already been studied (28), there is still a lack of knowledge in the field of intensive care nurses intention to leave and intention to stay. The intensive care area is a particularly stressful area (3) that requires special skills and therefore cannot be equated with normal wards. Therefore, we assume that factors other than organisational factors play a role in the study of intensive care nurses' turnover intention. Furthermore, the reasons for the intention to stay in intensive care have not yet been fully explored, but are of great importance for research and especially for practice due to the worldwide nursing shortage (16, 1, 29). Moreover, there are only a few investigations on the impact of the Covid-19 pandemic, especially in the intensive care sector. Most of them are cross correlational studies with a quantitative approach. The impact of the Covid-19 pandemic on turnover intention of intensive care nurses has been neglected in systematic reviews until now. We assume that the pandemic may have influenced intensive care workers' intention to leave (30, 31).

**Limitations**

We anticipate that generalizability will be an issue due to heterogeneity of objectives, study designs and instruments used in the included studies. As we have analysed secondary data, we also need to be aware of possible reporting bias.

**Ethical approvement**

Not applicable based on research of secondary data.

**Data availability**

Data are available from the corresponding author upon request.

**References**

1. WHO. State of the world's nursing 2020: investing in education, jobs and leadership. Geneva: World Health Organization; 2020.

2. Ferrer J, Boelle P-Y, Salomon J, Miliani K, L'Hériteau F, Astagneau P et al. Management of nurse shortage and its impact on pathogen dissemination in the intensive care unit. Epidemics 2014; 9:62–9.

3. Bruyneel A, Bouckaert N, Maertens de Noordhout C, Detollenaere J, Kohn L, Pirson M et al. Association of burnout and intention-to-leave the profession with work environment: A nationwide cross-sectional study among Belgian intensive care nurses after two years of pandemic. International Journal of Nursing Studies 2022; 137:104385.

4. Poon Y, Lin YP, Griffiths, P. Yong, K.K., Seah B, Liaw SL. A global overview of healthcare workers' turnover intention amid COVID-19 pandemic: a systematic review with future directions. Human Resources for Health 2022; 20(70):1–18.

5. Price J. The impact of turnover on the organization. Work and Occupations 1989; 16(4):461–73.

6. Lee, T. & Mowday, R. Voluntarily Leaving an Organization: An Empirical Investigation of Steers and Mowday's Model of Turnover. The Academy of Management Journal 1987; 30(4):721–43.

7. Mobley WH. Intermediate linkages in the relationship between job satisfaction and employee turnover. Journal of Applied Psychology 1977; 62(2):237–40.

8. Chegini Z, Asghari Jafarabadi M, Kakemam E. Occupational stress, quality of working life and turnover intention amongst nurses. Nursing in Critical Care 2019; 24(5):283–9.

9. van Dam K, Meewis M, van der Heijden BIJM. Securing intensive care: towards a better understanding of intensive care nurses' perceived work pressure and turnover intention. J Adv Nurs 2013; 69(1):31–40.

10. Heistad A, Goldsworthy S, Reilly S, Perez G. How do intensive work environments affect nurses' absenteeism and turnover intent? Appl Nurs Res 2022; 66:151608.

11. Liu Y-E, While A, Li S-J, Ye W-Q. Job satisfaction and work related variables in Chinese cardiac critical care nurses. Journal of Nursing Management 2015; 23(4):487–97.

12. Stone PW, Larson EL, Mooney-Kane C, Smolowitz J, Lin SX, Dick AW. Organizational climate and intensive care unit nurses' intention to leave. Crit Care Med 2006; 34(7):1907–12.

13. Schardt C, Adams MB, Owens T, Keitz S, Fontelo P. Utilization of the PICO framework to improve searching PubMed for clinical questions. BMC Med Inform Decis Mak 2007; 7:16.

14. Higgins JPT, editor. Cochrane handbook for systematic reviews of interventions: Second edition. Second edition. Hoboken NJ: Wiley-Blackwell; 2020. (Cochrane book series).

15. Xu G, Zeng X, Wu X. Global prevalence of turnover intention among intensive care nurses: A meta-analysis. Nursing in Critical Care 2021; (28):159–66.

16. WHO. The World Health Report 2000: Health Systems: Improving Performance. France: World Health Organization; 2000.

17. WHO. Working for health and growth: investing in the health workforce. Report of the High-Level Commission on Health Employment and Economic Growth 2016.

18. Khan, N., Jackson, D., Stayt, L. & Walthall, H. Factors influencing nurses' intentions to leave adult critical care settings. Nursing in Critical Care 2019; 24(1):24–32.

19. Liberati A, Altman DG, Tetzlaff J, Mulrow C, Gøtzsche PC, Ioannidis JPA et al. The PRISMA statement for reporting systematic reviews and meta-analyses of studies that evaluate health care interventions: explanation and elaboration. PLoS Med 2009; 6(7):e1000100.

20. Ziegler, A., Antes, G., & König, I. Bevorzugte Report Items für systematische Überscihten und Meta-Analysen: Das PRISMA-Statement. Deutsche Medizinische Wochenschrift 2011; (136):9–15.

21. Elm E von, Altman DG, Egger M, Pocock SJ, Gøtzsche PC, Vandenbroucke JP. Das Strengthening the Reporting of Observational Studies in Epidemiology (STROBE-) Statement. Notfall + Rettungsmedizin, Zeitschrift für präklinische und innerklinische Notfallmedizin 2008; 11(4):260–5.

22. Tong A, Sainsbury P, Craig J. Consolidated criteria for reporting qualitative research (COREQ): a 32-item checklist for interviews and focus groups. Int J Qual Health Care 2007; 19(6):349–57.

23. Hong, Q., Pluye, P., Fabriegues, S., Bartlett, G., Boardman, F., Cargo, M., Dagenais, P., Gagnon, M., Griffiths, F., Nicolau, B., O’Cathain, A., Rousseau, M. & Vedel, I. Mixed Methods Appraisal Tool (MMAT): version 2018 2018.

24. Griffeth RW, Hom PW, Gaertner S. A Meta-Analysis of Antecedents and Correlates of Employee Turnover: Update, Moderator Tests, and Research Implications for the Next Millennium. Journal of Management 2000; 26(3):463–88.

25. Price, J. & Mueller, C. A Causal Model of Turnover for Nurses. Academy of Management Journal 1981; 24(3):543–65.

26. MItchell, T., Holtom, B., Lee, T., Sablynski, C. & Erez, M. Why People Stay: Using Job Embeddedness to Predict Voluntary Turnover 2001; 44(6):1102–21.

27. Hom, Peter W., Allen, David G., Griffeth, Rodger W., editors. Employee Retention and Turnover; Why Employees Stay or Leave; First Edition. New York: Taylor & Francis; 2020.

28. Duffield CM, Roche MA, Homer C, Buchan J, Dimitrelis S. A comparative review of nurse turnover rates and costs across countries. J Adv Nurs 2014; 70(12):2703–12.

29. WHO. Working for Health Action Plan 2022-2030 2022.

30. Azoulay E, Pochard F, Reignier J, Argaud L, Bruneel F, Courbon P et al. Symptoms of Mental Health Disorders in Critical Care Physicians Facing the Second COVID-19 Wave: A Cross-Sectional Study. Chest 2021; 160(3):944–55.

31. Falk A-C, Nymark C, Göransson KE, Vogelsang A-C von. Missed nursing care in the critical care unit, before and during the COVID-19 pandemic: A comparative cross-sectional study. Intensive Crit Care Nurs 2022; 72:103276.
