# Supplementary material for: Turnover intention among intensive care nurses and the influence of the COVID-19 pandemic: a scoping review
Source: Hum Resour Health. 2025 May 15;23:23. doi: 10.1186/s12960-025-00992-7 (PMC12080060; doi:10.1186/s12960-025-00992-7)
Supplement: Supplementary file 2 — Additional file 2: Search strategy and search terms. [file 12960_2025_992_MOESM2_ESM.pdf]

Additional file 2 Search strategy and search terms

Search Tool Specification

**PCC**	**Description**	**Specification**	**Terms to search**
**Population**	Nurses	Nurse	Nurs*
**Concept**	Intention to leave (ITL), turnover intention, intention to quit, intention to stay (ITS) according to Hom et al. (2017).	Intention to leave and intention to stay	Turnover intent*, intent* to leave, intent* to quit, intent* to stay
**Context**	*Neonatal, pediatric, and adult Intensive Care Unit (ICU), Critical Care Unit in hospital setting*	Intensive Care or Critical Care	Intensive Care or Critical Care

Research Questions:

RQ 1: What factors impacting intensive and critical care nurses' intention to leave have been identified by the scientific literature so far?

RQ 2: What factors impacting intensive and critical care nurses' intention to stay have been identified in the scientific literature so far?

RQ3: What findings did the literature reveal regarding the impact of the COVID-19 pandemic on critical care nurses' intention to leave or stay?

Scoping review search strategy and search terms

Search strategy for factors that contribute to intensive and critical care nurses' intention to leave or stay

Database: Scopus

Search round	Query string
1	TITLE-ABS-KEY (turnover intent* AND critical care OR intensive care AND nurs*) AND PUBYEAR > 1999 AND PUBYEAR < 2023
2	TITLE-ABS-KEY (intent* to leave AND critical care OR intensive care AND nurs*) AND PUBYEAR > 1999 AND PUBYEAR < 2023
3	TITLE-ABS-KEY (intent* to quit AND critical care OR intensive care AND nurs*) AND PUBYEAR > 1999 AND PUBYEAR < 2023
4	TITLE-ABS-KEY (intent* to stay AND critical care OR intensive care AND nurs*) AND PUBYEAR > 1999 AND PUBYEAR < 2023

Database: Wiley

- Context Search: Abstract
- Custom range: 01/2000 – 12/2022

Search round	Query string
1	intent* to leave AND critical care OR intensive care AND nurs*
2	intent* to quit AND critical care OR intensive care AND nurs*
3	turnover intent* AND critical care OR intensive care AND nurs*
4	intent* to stay AND critical care OR intensive care AND nurs*

Database: PubMed

- Publication date: 2000 - 2022

Search round	Query
1	#1 “intention to leave”[tw] AND #2 “critical care” [tw] OR “intensive care” [tw] AND #3 “nurs*” [tw]
2	#1 “intent to leave”[tw] AND #2 “critical care” [tw] OR “intensive care” [tw] AND #3 “nurs*” [tw]
3	#1 “intention to stay”[tw] AND #2 “critical care” [tw] OR “intensive care” [tw] AND #3 “nurs*” [tw]
4	#1 “intent to stay”[tw] AND #2 “critical care” [tw] OR “intensive care” [tw] AND #3 “nurs*” [tw]
5	#1 “intention to quit”[tw] AND #2 “critical care” [tw] OR “intensive care” [tw] AND #3 “nurs*” [tw]
6	#1 “intent to quit”[tw] AND #2 “critical care” [tw] OR “intensive care” [tw] AND #3 “nurs*” [tw]
7	#1 “turnover intention”[tw] AND #2 “critical care” [tw] OR “intensive care” [tw] AND #3 “nurs*” [tw]
8	#1 “turnover intent”[tw] AND #2 “critical care” [tw] OR “intensive care” [tw] AND #3 “nurs*” [tw]

Database: APA PsycNet

Abstract: intent* to leave *OR* **Abstract**: intent* to quit *OR* **Abstract**: intent* to stay *OR* **Abstract**: turnover intent* *AND* **Abstract**: critical care *OR* **Abstract**: intensive care *AND* **Abstract**: nurs* *AND* **Peer-Reviewed Journals only** *AND* **Year**: 2000 *To* 2022

Database: Web of science

- Publication year: 2000 - 2022

Search round	Query
1	((AB=(intent* to leave)) AND AB=("intensive care" OR "critical care")) AND AB=(nurs*)
2	((AB=(intent* to quit)) AND AB=("intensive care" OR "critical care")) AND AB=(nurs*)
3	((AB=(intent* to stay)) AND AB=("intensive care" OR "critical care")) AND AB=(nurs*)
4	((AB=(turnover intent*)) AND AB=("intensive care" OR "critical care")) AND AB=(nurs*)
