# Supplementary material for: Turnover intention among intensive care nurses and the influence of the COVID-19 pandemic: a scoping review
Source: Hum Resour Health. 2025 May 15;23:23. doi: 10.1186/s12960-025-00992-7 (PMC12080060; doi:10.1186/s12960-025-00992-7)
Supplement: Supplementary file 4 — Additional file 4: Coding framework. [file 12960_2025_992_MOESM4_ESM.pdf]

Additional File 4 Coding framework

Theme of analysis	Description of theme	Overarching factors/ codes	Subcodes	Identified variables
1 Factors associated with ITL	Factors influencing the intention to leave among ICU nurses.	1.1 Organizational factos	1.1.1 Commitment and integration	Organizational commitment; Continuance commitment; Normative commitment; Affective commitment; Organizational integration
			1.1.2 Quality of delivered care	Ethical misconduct; Nonbeneficial treatment; Futile care; Ethical awareness; Incompetent care by physicians; No optimal care due to organizational burden; Quality of delivered Care; Inappropriate Treatment / Care; Missed Nursing Care; Nursing foundations for quality of care; Moral distress; Nurse participation in hospital affairs
			1.1.3 Organizational structure and work environment	Healthy work environment; Magnet status; Hospital profitability; Safe work environment; Full time vs. part time working; Shift working; Positive scheduling climate /Flexibility in schedule; Duty stressors; Organizational policy stressors; Social benefits; Salary; Work environment related stress; Staffing and resource adequacy; General Organizational perspective; Organization’s efforts to meet the needs of employees; Job promotion; Traumatic nature of work environment; Work overload
			1.1.4 Leadership	Autonomy; Relationship to Supervisors; Personal Feedback; Empowering leadership;

				Responsibility / Control; Involving in decision making process; Effective decision making; Nurse manager ability, leadership and support of nurses
			1.1.5 Professional collaboration and communication	Relationship with employees; Communication climate; Open interdisciplinary reflection in team; Participation and involvement; Interpersonal relations stressors; Teamwork; Communication; Collegial nurse-physician relations/communication
		1.2 Individual factors	1.2.1 Mental health and social reasons	Sleep quality; Depression; Personal accomplishment; Depersonalization; Emotional exhaustion; Negative Effect on family life; Low level of Resilience; Compassion fatigue; Anxiety; Personal reasons
			1.2.2 Socio-demographic characteristics	Marital status; Age
			1.2.3 Professionalism	Professional commitment; Knowledge skills; Career Advancement; Working experience
			1.2.4 Job satisfaction	Relational nursing job satisfaction; Extrinsic nursing job satisfaction; Intrinsic nursing job satisfaction
			1.2.5 Internal drivers	Job enjoyment; Meaningful recognition; Job pride; Motivation for work
2 Factors associated with ITS	Factors influencing the intention to stay among ICU nurses.	2.1 Organizational factors	2.1.1 Quality of delivered care	Quality of care

			2.2.2<br>Organizational structure and work environment	Rewarding system; Structural Empowerment; Salary; Benefits; Promotion; Operating procedures; Healthy workplace; Flexibility; Organizational factors
			2.2.3<br>Leadership	Supervision
			2.2.4<br>Professional collaboration and communication	Respectfulness & Teamwork; Co-workers; Communication
		2.2 Individual factors	2.2.1<br>Professionalism	Professional Development; Professionalism
			2.2.2<br>Internal drivers	Compassion satisfaction
3 Covid-19 related factors associated with ITL	Covid-19 related factors influencing the intention to leave among ICU nurses.	3.1 Organizational factors	3.1.1<br>Quality of delivered care	Moral distress, missed care
			3.1.2<br>Organizational structure and work environment	Staffing ressources
		3.2 Individual factors	3.2.1<br>Mental health and social reasons	Burnout, anxiety
